# The Diagnostic Scope of Sensor-Based Gait Analysis in Atypical Parkinsonism: Further Observations

**DOI:** 10.3389/fneur.2019.00005

**Published:** 2019-01-22

**Authors:** Heiko Gaßner, Cecilia Raccagni, Bjoern M. Eskofier, Jochen Klucken, Gregor K. Wenning

**Affiliations:** ^1^Department of Molecular Neurology, University Hospital Erlangen, Friedrich-Alexander University Erlangen-Nürnberg, Erlangen, Germany; ^2^Department of Neurology, Medical University of Innsbruck, Innsbruck, Austria; ^3^Machine Learning and Data Analytics Lab, Friedrich-Alexander University Erlangen-Nürnberg, Erlangen, Germany

**Keywords:** neurologic gait disorders, postural balance, biosensors, Parkinson disease, progressive supranuclear palsy, multisystem atrophy, gait analysis

## Abstract

**Background:** Differentiating idiopathic Parkinson's disease (IPD) from atypical Parkinsonian disorders (APD) is challenging, especially in early disease stages. Postural instability and gait difficulty (PIGD) are substantial motor impairments of IPD and APD. Clinical evidence implies that patients with APD have larger PIGD impairment than IPD patients. Sensor-based gait analysis as instrumented bedside test revealed more gait deficits in APD compared to IPD. However, the diagnostic value of instrumented bedside tests compared to clinical assessments in differentiating APD from IPD patients have not been evaluated so far.

**Objective:** The objectives were (a) to evaluate whether sensor-based gait parameters provide additional information to validated clinical scores in differentiating APD from matched IPD patients, and (b) to investigate if objective, instrumented gait assessments have comparable discriminative power to clinical scores.

**Methods:** In a previous study we have recorded instrumented gait parameters in patients with APD (Multiple System Atrophy and Progressive Supranuclear Palsy). Here, we compared gait parameters to those of retrospectively pairwise disease duration-, age-, and gender-matched IPD patients in order to address this new research questions. To this aim, the PIGD score was calculated as sum of the MDS-UPDRS-3-items “gait,” “postural stability,” “arising from chair,” and “posture.” Gait characteristics were evaluated in standardized gait tests using an instrumented, sensor-based gait analysis system. Machine learning algorithms were used to extract spatio-temporal gait parameters. Receiver Operating Characteristic analysis was performed in order to detect the discriminative power of the instrumented vs. the clinical bedside tests in differentiating IPD from APD.

**Results:** Sensor-based stride length, gait velocity, toe off angle, and parameters representing gait variability significantly differed between IPD and APD groups. ROC analysis revealed a high Area Under the Curve (AUC) for PIGD score (0.919), and UPDRS-3 (0.848). Particularly, the objective parameters stance time variability (0.841), swing time variability (0.834), stride time variability (0.821), and stride length variability (0.804) reached high AUC's as well.

**Conclusions:** PIGD symptoms showed high discriminative power in differentiating IPD from APD supporting gait disorders as substantial diagnostic target. Sensor-based gait variability parameters provide metric, objective added value, and serve as complementary outcomes supporting clinical diagnostics and long-term home-monitoring concepts.

## Introduction

Postural instability and gait difficulty (PIGD) are substantial motor symptoms of idiopathic Parkinson's disease (IPD) and even more represented in atypical Parkinsonian disorders (APDs), such as Multiple System Atrophy (MSA) and Progressive Supranuclear Palsy (PSP). Differentiating IPD from APD at early disease stages is challenging due to the overlap of the clinical core symptoms and the mild expression of atypical signs (red flags) characteristic for MSA or PSP (1). Many research attempts have been tried to find discriminators in early diagnostic procedures, mostly failing or not showing sufficient precision (2). However, since differential diagnosis between IPD and APD is of high clinical relevance, affecting therapeutic and diagnostic decisions, we aimed to identify objective bedside tests for the discrimination between both diagnoses.

Recently, a multicentre study involving 11 European MSA Study Group sites (2) showed the discriminative diagnostic value of a battery of classical clinician-based bedside tests including the presence of PIGD symptoms, evaluated using history taking and physical examination. They showed that patients with APD had larger PIGD related symptoms than IPD in early disease stages and also presented a more rapid progression to severe PIGD symptoms.

The clinical severity including PIGD in Parkinsonian disorders is commonly defined by semi-quantitative rating scales, such as the Unified Parkinson's Disease Rating Scale (UPDRS) (3) or the Movement Disorder Society Unified Parkinson‘s Disease Rating Scale (MDS-UPDRS) (4, 5). However, these established clinical rating scales are rater-dependent, non-metric, and lack objectivity. Thus, there is a need for more objective, quantitative and rater- independent measures that complement the established clinical examination of motor symptoms in IPD and APD (6–9). Objective sensor-based gait analysis technologies provide metric motor assessments, show increasing reliability and provide additional clinical information for diagnostics and therapy (10–12). However, the clinical relevance of this objective information of gait patterns has to be further evaluated, in particular in comparison to established clinical ratings. Furthermore, the diagnostic discriminative value of sensor-based gait parameters in differentiating APD from IPD patients is not well-known. In an initial cross-sectional study, our research group showed that sensor-based gait parameters revealed objective differences between APD and IPD patients (6). In our initial study, we did not differentiate between IPD subtypes with and without tremor, which is typically absent in APD patients. Thus, we now compared IPD patients with tremor (tremor-dominant subtype) and without tremor (akinetic-rigid subtype) with APD patients in a retrospectively pairwise-matched design to address the following aims (1) to evaluate whether sensor-based gait parameters allow the discrimination between APD from IPD patients, and (2) to investigate if instrumented, objective bedside tests have comparable discriminative power to clinical scores.

## Subjects and Methods

60 patients (IPD *n* = 40, APD *n* = 20) were enrolled in the outpatient clinics of the Department of Neurology at the Medical University Hospital Innsbruck, Austria, and the Department of Molecular Neurology at the University Hospital Erlangen, Germany. IPD (akinetic-rigid *n* = 20 [8 female, 12 male], tremor-dominant *n* = 20 [7 female, 13 male]) and APD (*n* = 20 [9 female, 11 male]) were defined according to standard diagnostic criteria for IPD, MSA, and PSP (13–15). The evaluation of the discriminative power of sensor-based gait analysis was performed on inertial sensor-based gait recordings from a previous study in these rare diseases (MSA, PSP) (6). In order to discriminate APD from IPD patients, we retrospectively pairwise matched to the APD patients (*n* = 20) IPD patients (*n* = 40) selected from a larger patient cohort (*n* = 406). For the analysis of this study, IPD patients were divided into two categories regarding their clinical presentation: akinetic-rigid (IPD-AR, *n* = 20) and tremor-dominant (IPD-TD, *n* = 20). Patients belonging to this larger cohort visited the Movement Disorders outpatient clinic of the Department of Molecular Neurology at the University Hospital Erlangen, Germany between July 2014 and March 2016 (12). Groups were matched by disease duration in order to compare the cohorts in a similar disease stage, as well as by age and gender for the purpose of controlling important confounders for gait analysis. In detail, pairwise matching means that a patient in the IPD-AR or IPD-TD group was selected when his/her age, gender, and disease duration optimally fitted to a patient in the APD group. Patient by patient were matched with this method. In case, age or disease duration did not fit exactly, the IPD patient with characteristics most closely to the APD patient was chosen. In the very rare case that there were two or more patients in the larger cohort with exactly the same age, gender and disease duration, we have chosen one patient at random. During the matching process, *n* = 5 APD patients with very short disease duration but severe motor impairment were lost from our previous analysis (6) since there were no patients in the IPD groups that could be matched to. Although we did not match for global H&Y stage, this measure should not differ substantially between groups in order to answer this research question correctly. Consequently, *n* = 20 patients per group were included in the present analysis with matched cohorts.

APD patients (MSA-p *n* = 11, PSP *n* = 9) were not divided into subgroups (MSA, PSP) due to the small sub-cohort size in these rare diseases. Moreover, our own previous data (6) and others (2) did not show any differences in motor scores between MSA and PSP (6). Exclusion criteria consisted of non-PD related gait impairments (e.g., spinal or orthopedic surgery), spasticity, stroke, neuropathy, myelopathy, hydrocephalus, and severe dementia. IPD patients were investigated in stable ON medication without presence of motor fluctuations and APD patients were assessed on regular therapy. The study was approved by the local ethics committee (IRB-approval-Re.—No. 4208, 21.04.2010, Medical Faculty, Friedrich-Alexander-University Erlangen-Nürnberg, Germany, IRB-approval-Re.—No. 0365, 27.04.2015, Medical Faculty Innsbruck, Austria), and all participants gave written informed consent according to the Declaration of Helsinki.

### Clinical Ratings and Sensor-Based Gait Analysis

IPD, MSA-p and PSP patients were rated by MDS-UPDRS-3 (4). PIGD score was calculated as defined (16) as sum of MDS-UPDRS-3 items 3.9 (arising from chair), 3.10 (gait), 3.12 (postural stability), and 3.13 (posture). Furthermore, MSA-p patients were scored using UMSARS (17) and PSP patients were assessed by PSP-RS (18).

A detailed description of sensor-based gait signatures and their association to clinical scales is presented in our previous work (6). Gait characteristics were evaluated in standardized gait tests using an instrumented, sensor-based gait analysis system. This system consists of wearable SHIMMER 2 sensors (Shimmer Research Ltd., Dublin, Ireland) laterally attached to the posterior lateral portion of both shoes. Gait signals were recorded within a (tri-axial) accelerometer range of ±6 g (sensitivity 300 mV/g), a gyroscope range of ±500 degree/sec (sensitivity 2 mV/degree/sec), and a sampling rate of 102,4 Hz. Sensor signals were transmitted via Bluetooth® to a tablet computer and stored for subsequent data analysis (19). Inertial sensor data was processed using a machine learning algorithm for calculating clinically relevant spatiotemporal gait parameters (e.g., stride length, gait velocity, maximum toe clearance, gait variability parameters) (20, 21). Participants performed standardized overground walking tests on a 10 m long corridor in the hospital in self-chosen walking speed. Only straight strides were automatically detected by the stride detection algorithm (20) and used for gait parameter calculations as described (21). Calculated gait velocity, stride length, cadence, and maximum toe clearance were normalized to the height of the participants.

### Statistical Analysis

An one-way ANOVA was used to verify differences between variables (age, disease duration, levodopa equivalent daily dose (LEDD), MDS-UPDRS-3, H&Y, PIGD, gait parameters), and diagnosis (IPD-AR, IPD-TD, APD). One-way ANOVA was followed by Bonferroni *post-hoc* multiple comparison procedure to detect differences between patient groups. Gender differences were tested using chi-squared test. MDS-UPDRS-3, PIGD, and the four gait parameters with the largest *F*-values (gait variability parameters) were included in receiver operating characteristic (ROC) analysis. To evaluate the diagnostic effect size of bedside tests, ROC curves were plotted, and the area under the curve (AUC), as well as sensitivity and specificity for each parameter were calculated. Youden's statistics were used to identify the cut-off score of each parameter that shows the most effective discriminative value between IPD and APD groups (22).

## Results

**Figure 1:**
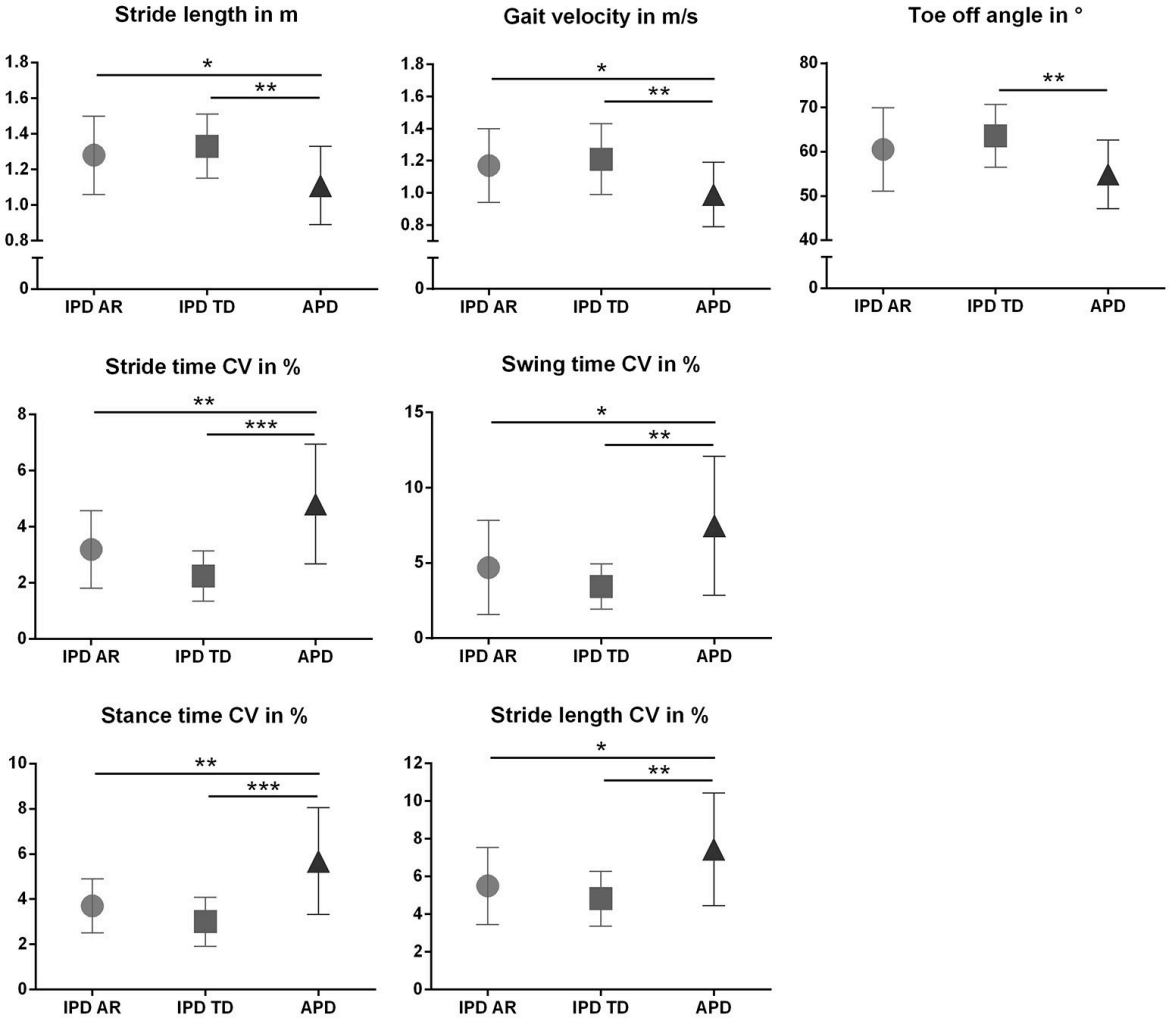
Spatiotemporal gait parameters (Mean ± SD) in patients with atypical Parkinson disorders (APD), and age and gender matched patients with idiopathic Parkinson‘s disease (IPD) separated in akinetic-rigid (IPD-AR) and tremor-dominant (IPD-TD) subtype. CV, Coefficient of Variance. ^*^p < 0.05, ^**^p < 0.01, ^***^p < 0.001.

The discriminative power of the clinical and instrumented bedside tests to differentiate between APD and IPD was evaluated in patient cohorts pairwise matched by disease duration, age, and gender. Patient characteristics are presented in Table 1, and sensor-based gait parameters in Table 2 and Figure 1. As expected, cohorts significantly differed in PIGD (APD 7.4 ± 1.8, IPD-AR 4.4 ± 1.6, and IPD-TD 3.3 ± 1.7; *F* = 31.074, *P* = 0.000) and UPDRS-3 (APD 38.4 ± 11.4, IPD-AR 22.1 ± 9.0 and IPD-TD 24.1 ± 9.6; *F* = 15.598, *P* = 0.000). Gait velocity was significantly reduced in APD patients (0,99 ± 0.20 m/s) compared to IPD patients (IPD-AR 1.17 ± 0.20 m/s, IPD-TD 1.21 ± 0.20 m/s; *F* = 6.016, *P* = 0.004). Similar results were obtained for stride length (APD 1.11 ± 0.20 m, IPD-AR 1.28 ± 0.20 m and IPD-TD 1.33 ± 0.20 m; *F* = 5.953, *P* = 0.004). APD patients demonstrated a decreased toe off angle (54.9 ± 7.8°) compared to IPD patients (IPD-AR 60.5 ± 9.5°, IPD-TD 63.6 ± 7.1°; *F* = 5.769, *P* = 0.005). Gait variability parameters that reflect impairment of dynamic postural control have shown to be increased in APD patients. Swing time variability was significantly increased in APD (7.48 ± 4.6%; *F* = 7.687, *P* = 0.001) compared to IPD-AR (4.70 ± 3.1%) and even more increased in comparison to IPD-TD (3.44 ± 1.5%). Stride time variability showed similar patterns (IPD AR: 3.19 ± 1.4, IPD-TD: 2.24 ± 0.9, APD: 4.81 ± 2.0; *F* = 13.948, *P* = 0.000). Stance time variability was significantly increased in APD patients (5.69 ± 2.4; *F* = 14.073, *P* = 0.000) compared to IPD-AR (3.70 ± 1.2) and IPD-TD (3.01 ± 1.1). Similar results were detected for stride length variability (APD 7.45 ± 3.0%, IPD-AR 5.50 ± 2.1%, IPD-TD 4.83 ± 1.4%; *F* = 7.183, *P* = 0.002). For heel strike angles, stride time, swing time, stance time, step cadence, and maximum toe clearance we did not observe significant differences between the three groups. Within the two IPD subpopulations no significant differences were observed in any of the spatio-temporal gait parameters.

**Table 1:** Patient characteristics.

	**IPD AR**	**IPD TD**	**APD**	***P***	**Bonferroni** ***post-hoc***		
					**AR vs. APD**	**AR vs. TD**	**APD vs. TD**
***n***	**20**	**20**	**20**			
Age (y)	67.7 ± 9.7	65.6 ± 8.5	65.8 ± 8.3	0.706	1.000	1.000	1.000
Gender (male:female)	12:08	13:07	11:09	0.812^*^		
Disease duration (y)	6.6 ± 3.6	6.4 ± 3.2	5.2 ± 2.9	0.340	0.528	1.000	0.735
H&Y	2.9 ± 0.4	2.6 ± 0.4	3.1 ± 0.4	**0.002**	0.449	0.076	**0.001**
MDS-UPDRS-3	22.1 ± 9.0	24.1 ± 9.6	38.4 ± 11.4	**0.000**	**0.000**	1.000	**0.000**
PIGD	4.4 ± 1.6	3.3 ± 1.7	7.4 ± 1.8	**0.000**	**0.000**	0.122	**0.000**
LEDD (mg/d)	591.6 ± 386.9	463.3 ± 378.4	712.3 ± 401.9	0.139	0.993	0.905	0.143

*One-way ANOVA followed by Bonferroni post-hoc test*,

* *Chi-squared test, Significance level p < 0.05. Bold numbers indicate significance*.

*H&Y, Hoehn and Yahr disease stage, MDS-UPDRS-3 (motor examination); LEDD, Levodopa equivalent daily dose; PIGD, Postural Instability and Gait Difficulty Score; IPD AR, akinetic-rigid subtype of Parkinson's disease; IPD TD, tremor-dominant subtype of Parkinson's disease; APD, Atypical Parkinsonian Disorders including MSA, Multiple system Atrophy; PSP, Progressive Supranuclear Palsy*.

**Table 2:** Sensor-based gait parameters in IPD and APD patients, categorized by gait characteristics defined by (12).

	**IPD AR**	**IPD TD**	**APD**	***P***	**Bonferroni** ***post-hoc***		
					**AR vs. APD**	**AR vs. TD**	**APD vs. TD**
***n***	**20**	**20**	**20**			
**SHORT STEPS AND BRADYKINESIA**							
Stride length (m)	1.28 ± 0.2	1.33 ± 0.2	1.11 ± 0.2	**0.004**	**0.041**	1.000	**0.005**
Gait velocity (m/s)	1.17 ± 0.2	1.21 ± 0.2	0.99 ± 0.2	**0.004**	**0.035**	1.000	**0.005**
Stride time (s)	1.10 ± 0.1	1.10 ± 0.1	1.14 ± 0.1	0.609	1.000	1.000	1.000
Stance time (%)	64.7 ± 1.5	64.8 ± 2.3	65.8 ± 2.0	0.143	0.255	1.000	0.272
Swing time (%)	35.2 ± 1.5	35.2 ± 2.3	34.2 ± 2.0	0.157	0.278	1.000	0.297
Cadence (strides/min)	54.8 ± 5.2	54.8 ± 5.0	53.5 ± 5.6	0.672	1.000	1.000	1.000
**SHUFFLING OF GAIT**							
Angle HS (°)	8.20 ± 5.6	8.55 ± 5.9	8.78 ± 6.5	0.955	1.000	1.000	1.000
Angle TO (°)	60.5 ± 9.5	63.6 ± 7.1	54.9 ± 7.8	**0.005**	0.101	0.740	**0.004**
Max. TC (cm)	6.88 ± 2.9	7.30 ± 2.3	7.10 ± 3.2	0.893	1.000	1.000	1.000
**REGULARITY OF GAIT**							
Stride time CV (%)	3.19 ± 1.4	2.24 ± 0.9	4.81 ± 2.0	**0.000**	**0.005**	0.176	**0.000**
Swing time CV (%)	4.70 ± 3.1	3.44 ± 1.5	7.48 ± 4.6	**0.001**	**0.032**	0.709	**0.001**
Stance time CV (%)	3.70 ± 1.2	3.01 ± 1.1	5.69 ± 2.4	**0.000**	**0.001**	0.588	**0.000**
Stride length CV (%)	5.50 ± 2.1	4.83 ± 1.4	7.45 ± 3.0	**0.002**	**0.027**	1.000	**0.002**

*One-way ANOVA followed by Bonferroni post-hoc test. Significance level p < 0.05. Bold numbers indicate significance. IPD AR, akinetic-rigid subtype of Parkinson's disease; IPD TD, tremor-dominant subtype of Parkinson's disease; APD, Atypical Parkinsonian Disorders; CV, Coefficient of Variance; HS, heel strike; TO, toe off; Max. TC, maximum toe clearance*.

**Figure 2:**
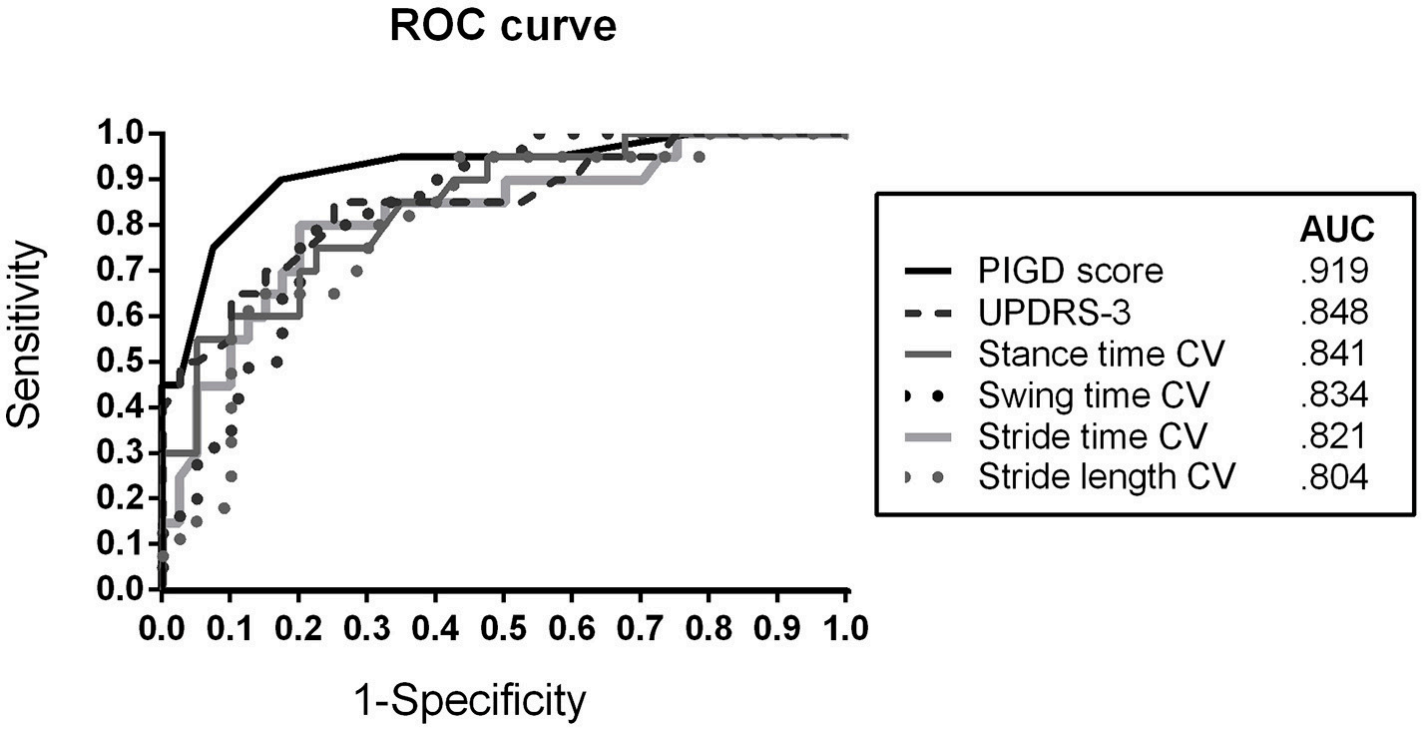
Receiver operating characteristic (ROC) curve for discriminating APD patients from IPD (AR + TD) patients using clinical rating scores (MDS-UPDRS-3, PIGD) and spatiotemporal gait variability parameters CV, Coefficient of Variance; APD, Atypical Parkinsonian Disorders; AR, akinetic-rigid; TD, tremor-dominant; PIGD, Postural Instability and Gait Difficulty Score; AUC, Area under the curve.

In order to evaluate the discriminative power of the bedside tests, ROC analysis was performed. Figure 2 presents the ROC curve and AUC values for differentiating IPD (IPD-AR + IPD-TD) from APD patients. The PIGD score showed an AUC of 0.919 (sensitivity 90.0%; specificity 82.5%), followed by MDS-UPDRS-3 with an AUC of 0.848 (sensitivity 85.0%; specificity 75.0%). The gait parameter with the largest discriminative value was stance time variability reaching an AUC of 0.841 (sensitivity 75.0%; specificity 77.5%). Similar results were shown for stride time variability (AUC = 0.821; sensitivity 80.0%; specificity 80.0%), swing time variability (AUC = 0.834; sensitivity 80.0%; specificity 77.5%), and stride length variability (AUC = 0.804; sensitivity 95.0%; specificity 57.5%). Youden's statistics revealed the following cut-off scores: PIGD ≥ 5.5, MDS-UPDRS-3 ≥ 26.5, stance time variability ≥ 4.1%, stride time variability ≥ 3.3%, swing time variability ≥ 4.4%, and stride length variability ≥ 5.2%.

**Figure 3:**
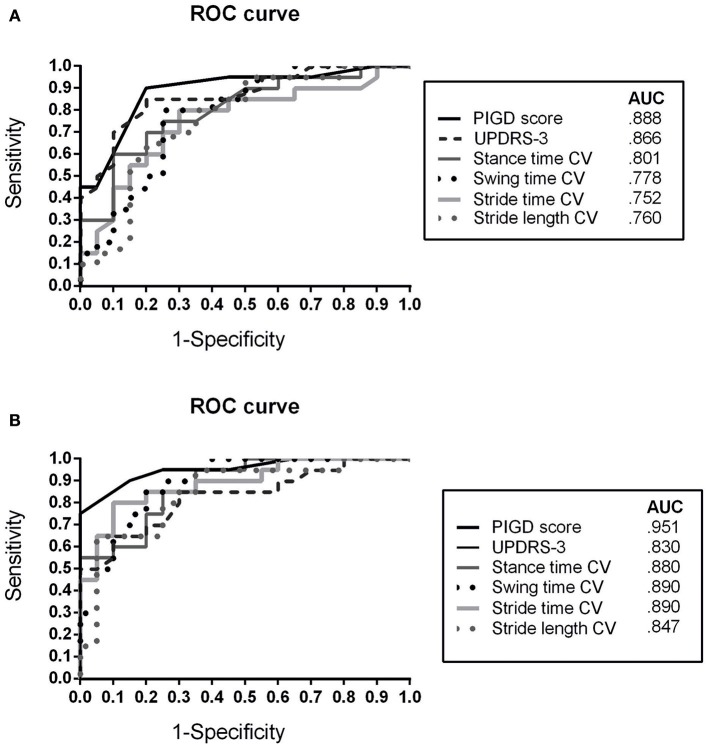
Receiver operating characteristic (ROC) curve for discriminating APD patients from **(A)** IPD AR and **(B)** IPD TD patients using clinical rating scores (MDS-UPDRS-3, PIGD) and spatiotemporal gait variability parameters CV, Coefficient of Variance; APD, Atypical Parkinsonian Disorders; AR, akinetic-rigid; TD, tremor-dominant; PIGD, Postural Instability and Gait Difficulty Score; AUC, Area under the curve.

The ROC analyses for discriminating between APD and IPD subgroups (APD vs. IPD-AR, APD vs. IPD-TD) showed similar results (Figure 3).

## Discussion

The aim of this study was to evaluate whether sensor-based gait parameters are able to discriminate APD from IPD patients, and to investigate if instrumented, objective bedside tests have comparable discriminative power to classical clinical bedside tests. Our findings revealed that instrumented gait parameters stride length, gait velocity, TO angle, and gait variability parameters significantly differed between matched APD and IPD patient cohorts. Moreover, sensor-based gait variability parameters discriminated between IPD and APD patients similarly to established clinical scores (PIGD, UPDRS-3).

Early differential diagnosis between IPD and APD would be a substantial advantage for the treatment of APD patients, with tremendous impact on clinical care and research. In order to compare clinical and instrumented bedside tests for their diagnostic value in differentiating APD and IPD patients, we pairwise matched our patient cohorts (IPD-AR, IPD-TD, and APD) by disease duration as well as by age and gender as important confounders for gait analysis. As expected, APD patients showed larger MDS-UPDRS-3 and PIGD scores compared to IPD patients with comparable disease duration reflecting more severe global motor and locomotor impairment as assessed by classical clinical bedside tests (2). The PIGD score as a gait and balance related subscore of the MDS-UPDRS-3 showed larger discriminative value between APD and IPD patients than the global assessment of motor impairment by the MDS-UPDRS-3 (Figure 2). This result confirmed that in particular gait and postural stability features are specific characteristics in APD at comparable disease duration and thus, should be specifically considered in the differential diagnostic workup of APD and IPD. Likewise, PIGD symptoms correlate with disease progression, prognosis, and risk of falls in IPD (23–25), and thus should be used as clinical marker for long-term monitoring. Clinical scores (MDS-UPDRS-3, PIGD) presented the highest discriminative value in this analysis indicating the essential and undoubted value of established clinical examinations to evaluate gait and balance deficits.

However, large interrater variability has been shown for the “Pull Test” item of the PIGD score (26), and also the global evaluation of gait characteristics assessed by the clinical bedside test UPDRS depends on rater-experience and does not provide metric data of disease specific gait deficits (27). Thus, instrumented beside tests provide objective gait parameters that have the potential to support the clinical evaluation of gait by more granular, additional information on gait features (8, 9). Indeed, wearable sensors have already shown to detect short steps and shuffling of gait in IPD patients using objective, metric gait variables provided by inertial sensors and machine learning algorithms (12).

The instrumented gait analysis revealed that matched IPD and APD cohorts differed in stride length, gait velocity, toe off angle, and gait variability parameters meaning that APD patients walk with shorter and slower steps, more progressed shuffling of gait and are impaired in gait regularity. Interestingly, regularity of gait reflected by gait variability parameters showed the most characteristic differences of all sensor-based gait variables between both matched cohorts based on F-values. In the ROC analysis, gait variability presented high discriminative value with similar AUC's compared to classical clinical scores. In particular, for the differentiation between IPD-TD and APD, gait variability parameters reached larger AUC values compared to UPDRS-3 suggesting that regularity of gait may serve as sensitive biomarker in discriminating APD from IPD-TD patients. Increased gait variability indicates that APD patients have a more pronounced impairment of quality of gait reflecting deficits in dynamic postural control and increased risk of falling (28). This aspect is interesting for further characterization of specific sensor-based gait profiles of APD patients.

During the diagnostic workup, additional clinical signs (so called “plus signs” or “red flags”) are indicative for the development of an atypical form including rapid progression of gait impairment, early postural instability, and ataxia symptoms (1, 29). During the disease course, a progression of postural instability is reflected by larger gait variability (30). This impaired gait regularity serves as an objective marker for deficits in dynamic postural control (12) and may indicate the early occurrence of postural instability in APD. It has further been shown that gait disorders in patients with cerebellar ataxia are correlated with increased gait variability (31). This is in line with our findings and suggests that gait variability parameters should be focused for supporting the differential diagnosis of APD. Furthermore, parameters representing gait variability have been reported as quantitative measures strongly linked to deficits in gait and postural stability in IPD and serve as indicator for fall risk (32–34). Therefore, gait variability parameters play a major role in evaluating dynamic postural control in APD patients since falls are mostly present in these diseases. Detecting deficits in dynamic postural stability early in the disease course has the substantial advantage to adapt therapy and possibly prevent falls.

These results suggest that gait variability parameters provide added value in discriminating APD from IPD patients, therefore substantially support the clinical rating of motor symptoms and indicate deficits of dynamic postural control. Sensor-based movement analysis provides several metric parameters reflecting motor impairment, and has the potential to objectively track disease progression in longitudinal settings (e.g., in tele-monitoring applications) (7, 8). Future studies should aim to follow this research question of finding appropriate biomarkers to discriminate APD from IPD patients early in the disease course.

For the differential diagnosis between APD and IPD, Youden's indices of ≥ 5.5 for PIGD and ≥ 26.5 for MDS-UPDRS-3 indicate that it is most likely to identify an APD patient if the clinical score is equal or higher the mentioned cut-off score. In a similar way, cut-off scores for quantitative, sensor-based gait variability parameters were defined in order to identify APD patients using objective measures. Future studies should re-evaluate these cut-off scores in larger cohorts and earlier in the disease stage.

## Limitations

A few limitations of our study have to be mentioned. Our patient cohorts had an average disease duration of 5–6 years. For future studies it would be preferable to investigate patients more early in the disease course which is important for differential diagnosis but very challenging. Due to lack of statistical power when analyzing MSA and PSP patients separately, we combined these patients in one APD group. Furthermore, we did not perform post-mortem neuropathological diagnosis confirmation, therefore a certain rate of possible misdiagnosis has to be considered. To minimize this problem, clinical diagnosis was performed by movement disorder specialists according the diagnostic criteria for IPD and APD. Thus, the clinical tests and scores we examined here mainly reflected the subjective decisions of experts, based on overall clinical presentation.

## Conclusion

In conclusion, our data demonstrate that sensor-based gait parameters complement clinical diagnostic workup by providing metric, objective information as easy performable instrumented bedside tests. In particular, gait variability parameters discriminated between IPD and APD patients similarly to established clinical scores. Besides the discriminative power, sensor-based gait analysis provides more granular and comprehensive information on gait impairment, and therefore complements neurological examination in clinical practice. Future studies should aim to further investigate the discriminative value of sensor-based gait parameters in early disease stages.

## Disclosure

This was not an industry supported study. This work was performed at the Department of Molecular Neurology, University Hospital Erlangen, Erlangen, Germany, and at the Department of Neurology, Innsbruck Medical University, Innsbruck, Austria.

## Author Contributions

HG, CR, JK, GW: Design and conception; HG, CR, JK: Organization and execution; HG: Statistical analysis; HG, CR: Writing first draft of the manuscript; BE, JK, GW: Review and critique.

### Conflict of Interest Statement

HG, BE, and JK received institutional research grants from the Emerging Field Initiative of the Friedrich Alexander-University Erlangen-Nürnberg (EFI Moves, 2 Med 03), from the Bavarian State Ministry for Education, Science and the Arts, Munich, Germany (MotionLab@Home, E|Home Center), from the Bavarian Ministry of Economic Affairs and Media, Energy and Technology, Germany (Medical Valley Award 2016, Risk-e-Gait), and from the European Institute of Innovation and Technology (EIT Health, “MoveIT”, EIT Digital, “vital@home”). CR declares a research grant from the MSA Coalition and from the Austrian Parkinson's disease. BE holds ownerships of Portabiles HealthCare Technologies GmbH and Portabiles GmbH, received compensation and honoraria from serving on scientific advisory boards for Abbvie GmbH, Adidas GmbH, Bosch Sensortec GmbH, and ST Sportservice GmbH. Further, he gratefully acknowledges the support of the German Research Foundation (DFG) within the framework of the Heisenberg professorship programme (grant number ES 434/8-1). GW reports receiving consulting and/or lecture fees from Affiris, Astra Zeneca, Boehringer Ingelheim, Ever Pharma, Lundbeck, Neuropore, Orion, and UCB as well as grant support from Medical University Innsbruck, Oesterreichische Nationalbank, FWF Austrian Science Fund, US MSA Coalition, Affiris, Astra Zeneca, and Boehringer Ingelheim. JK holds ownerships of Portabiles HealthCare Technologies GmbH and Portabiles GmbH, received compensation, and honoraria from serving on scientific advisory boards for LicherMT GmbH, Abbvie GmbH, UCB Pharma GmbH, GlaxoSmithKline GmbH & Co. KG, Athenion GmbH, and Thomashilfen GmbH; as well as lecturing from UCB Pharma GmbH, TEVA Pharma GmbH, Licher MT GmbH, Desitin GmbH, Abbvie GmbH, Solvay Pharmaceuticals, and Ever Neuro Pharma GmbH.
